# Examining Gender Differences in Aggression as a Predictor of Anxiety, Depression, and Suicide in a Cross‐Sectional French Sample

**DOI:** 10.1002/hsr2.70359

**Published:** 2025-01-13

**Authors:** Sylvia Martin

**Affiliations:** ^1^ Center for Research and Bioethics Uppsala University Uppsala Sweden; ^2^ Private Practice‐Psycho‐TCCE Montpellier France

**Keywords:** aggression, anxiety, depression, gender, suicide risk, women

## Abstract

**Background and Aims:**

The expected outcomes of the Covid‐19 pandemic have a supposedly worsened impact on mental health and suicidal risk. Traditionally, males were supposed to have heightened Aggression and impulsivity in stressful times. We aim to measure the actual differences that existed during the COVID‐19 pandemic across male and female participants.

**Methods:**

An overall number of 288 females/95 males (mean age: men = 34.97; women = 32.90 years) participants were recruited. The protocol included questionnaires about suicidal risk, anxiety, depression, impulsivity, aggression, hopelessness, and demographics.

**Results:**

Differences exist in the sense of loneliness, the number of persons being isolated with, anxiety, and several impulsivity subdimensions. A correlation existed for both men and women for anxiety and depression, revealing the impact of aggression. Suicide men's scores correlated with anxiety and depression, lack of premeditation, and hopelessness. Women also had such correlation, except for lack of perseverance, social dimensions, and aggression. Analyses reveal the predictive impact of hopelessness and aggression on anxiety, depression, and suicidal risk predominantly in females.

**Conclusion:**

Our results contradict common ideas that men have heightened aggression and impulsivity. Further research needs to address aggression issues.

## 1 Introduction

During the coronavirus disease (COVID‐19) outburst in France in 2020, experts feared that vulnerable populations could suffer from anxiety and depression issues. Data gathered evidence that psychological distress was a worsened factor in general health considerations. During previous outbreaks from severe acute respiratory syndrome (SARS) in 2003 to Ebola and Zika virus respectively in 2014 and 2016, community members perceived an increase in stress due to media consumption, economic insecurity, fear of healthcare system reliability, traumatic experiences, isolation, and fear of the disease [1, 2, 3]. Research also showed poorer mental health with short‐term and long‐term effects on psychiatric disorders like depression and anxiety [1, 2, 3]. The expected outcomes of such catastrophes like the Covid‐19 pandemic have a supposed worsened impact on women. In 2020, Connor et al. [4] addressed this specific risk. They stated that “*[…] the […] gender differences in stress experience and exposure are likely exacerbated by pandemics. when insults to mental wellbeing occur, they are added to women's baseline stress and feelings of disempowerment, increasing the risk of mental health disorders*.” Nevertheless, other research pointed out many more factors of vulnerability for women's mental health issues, like age, lower psychopathological levels (mild levels of psychopathology), being pregnant, and having a chronic disease. Women are also vulnerable due to their high vulnerability from intimate partner violence [5, 6]. Overall, the metanalysis was inconclusive and revealed a need to run further research to allocate resources efficiently [7]. On the other side of the classical gender spectrum, males were supposedly more impulsive and aggressive in times of crisis [8, 9]. Another suspicion was that women would suffer more from anxiety and depression. Mirroring that, systematic literature offered results proving that men had aggression issues whereas females struggled with stress and mental health symptom [10]. Nevertheless, a meta‐analysis also noted the neglect of sex‐ and gender‐specific evaluation of aggression‐related disorders [11].

### 1.1 Aggression During COVID 19 Pandemic

In 2020, Sadiq et Shaphiq [12] examined differences in aggression in married couples. Aggression from men was more focused on physical aggression than from women involved in verbal aggression. Nevertheless, concomitant factors like anger and hostility were present in both genders. In 2021, Thomson et al. [13] drew an interesting conclusion on the relationship between fear and aggression reactions in both genders. Independently of sex, reactive aggression was related to heightened sympathetic nervous system's fear reactivity and hypersensitivity to fear. In 2021, Mendez et al. [14] also questioned the aggression related to psychopathology across gender, concluding that aggression intervention should be tailored to gender after exploring the gender's role in the psychopathology‐aggression relationships (e.g., internalizing and externalizing behaviors). During the pandemic, Aggression in relation with alcohol use was measured to be heightened for women compare to men participants [15].

### 1.2 Suicidal Risk, an Anticipated Consequence

No matter gender, specialists from the psychiatric field expected increased suicidal rates. Farooq et al., in 2021 [16], retrieve risk factors: low social support, high physical and mental exhaustion and poorer self‐reported physical health, sleep issues, quarantine and exhaustion, loneliness, and mental health difficulties. The overall suicidal ideations being higher than in previous periods before the pandemic could result in heightened suicidal rates. Indeed, even of not experiencing depression or anxiety, individuals with heightened alexithymia during the pandemic [17] could have been at higher risk of suicide and self‐harm [18]. Dubé et al., in 2021 [19], appealed to this consideration by revealing the little‐known factors that could influence suicidal ideation: younger age, and women from a democratic country. They exhorted policymakers and professionals to take measures based on age, gender, and geopolitics to tackle suicidal risk during COVID‐19 pandemic. Even if later research proved that there was no significant increase in suicidal rates worldwide [20], there was an overall concern at this time for suicidal factors to be increased.

The aim of the paper will compare gender differences in mental health measures and aggression and impulsivity specifically during the COVID‐19 pandemic. If adequate, causal relationships will be explored.

## 2 Methods

### 2.1 Participants

A total number of 383 respondents (286 females and 95 males) were recruited to an online questionnaire using Google Forms during the second month of isolation in France (from April to May 2020 representing the quarantine period that started in March and was progressively “lightened” starting mid‐May 2020) representing the participant whom fully answered the online questionnaire. In case a participant was screened but did not completed all questionnaire, the results were not saved and therefore no data for subject not included due to non‐completion of the form are not available. According to domestic regulations (“Jardé Law” RIPH3 for non‐interventional research in the *Code of Public Health* Articles L1121‐1 à L1128‐12), ethical committee approval was deemed unnecessary. All participants received information about the purpose of the study, its objectives, participation procedures, potential benefits and risks, rights for withdrawing of participation, data management and security, absence of financial compensation, explanation about responsabilities and were offered to ask any question via direct email contact to the investigator. After confirming that they have red and understood the information by validating the form, they were providing online informed consent to participate in this experiment and for publication of the subsequent results according to Helsinki's recommendation and General Data Protection Regulation (GDPR). They responded to clinical scales and demographic data for approximately 20–30 min. They declare their correspondence to inclusion and exclusion criteria as a sworn statement. Exclusion criteria for both groups were: (a) known neurological disease, and (b) developmental disability (c) be proficient in the French language.

### 2.2 Measures

In a recent transdiagnostic understanding of psychiatric disorder, we used measures of the dynamics at stake (impulsivity, aggression, control, openness) and no pathognomy scales [21, 22]. To address the general population's potential psychological disorder, validated measures were used in clinical psychology research in a wide range of disorder research (see methods sections with corresponding application fields for each scale: Beck Hopelessness Scale (BHS), Suicide Behavior Questionnaire Revised (SBQ‐R), Hospital Anxiety and Depression Scale (HADS) [23, 24, 25]) and transdiagnostic dimension measures for the impulsive behavior scale (Negative Urgency, (lack of) Premeditation, (lack of) Perseverance, Sensation Seeking, and Positive Urgency) (UPPS) and Aggression questionnaire (AQ12) (see [26, 27, 28, 29, 30, 31]).

### 2.3 BHS, Beck Hopelessness Scale

The hopelessness scale, in its French translation by Bouvard et al., 1992 [32], evaluated pessimism and cognitive beliefs about the future, indirectly reflecting suicidal intentions. Items are to be completed via a binary quotation “true/false,” the total score varies from 0 to 20. Cronbach's alpha is 0.72.

### 2.4 SBQ‐R, Suicidal Behavior Questionnaire‐ Revised

The 4‐item questionnaire assessing suicidal behaviors. SBQ‐R is one of the only tools asking about future anticipation of suicidal thoughts or actions and past and present ones and includes a question about lifetime experiences about suicidal (ideations, plans and attempts). French validation from Potard et al., 2014 [33] has the same structure as the original version with a total score ranging from 3 to 18. A total score of 7 or higher indicates a significant risk of suicidal behavior. Cronbach's alpha for the SBQ‐R items was 0.80. To date no French validation in adult's population is available. Even if initially validated in an adolescent sample, the SBQ‐R showed good properties across cultures in adults’ populations [34, 35, 36].

### 2.5 HADS, Hospital Anxiety and Depression Scale

HADS– in its French translation from Friedman et al. released in 2001 [37]‐ identifies the presence (possible and probable) of anxiety disorders and depression from a set of 14 questions. The maximum score is 21. It was divided into an anxiety subscale (HADS‐A) and a depression subscale (HADS‐D), containing seven intermingled items. This scale used a cut‐off of 9 or more for the depression subscale and 11 or more for the anxiety subscale in its original validation but a recognized unique cut‐off at 8 has also proven its relevance to detecting pathological levels of both psychological distress dimensions [38, 39]. The reliability factors Cronbach's alpha was: 0.67 for Anxiety and 0.79 alpha for depression.

### 2.6 AQ12, Aggression Questionnaire 12

The aggression questionnaire French translation from Genoud and Zimmerman [40] contains 29 items that assess four dispositional dimensions of aggression: physical aggression, verbal aggression, anger, and hostility. It can be used as a single score, adding all the dimensions. This latest version includes 12 items (self‐reported behavior and feelings) using a Likert scale from 1 (Not at all like me) to 6 (Completely like me). Cronbach's alpha coefficient of the whole questionnaire was 0.80. This version did not specify gender differences but the sample for validation in French was 34% men, 66% female.

### 2.7 UPPS‐P Impulsive Behavior Scale (Negative Urgency, (Lack of) Premeditation, (Lack of) Perseverance, Sensation Seeking, and Positive Urgency

The French translation [41] of this self‐report scale composed of 20 items assessing four factors of impulsivity: (a) urgency (negative and positive), (b) lack of premeditation; (c) lack of perseverance; (d) sensation seeking was used. Positive urgency assesses the impulsivity level due to positive emotion. Negative urgency evaluates impulsivity due to negative emotions. The respective Cronbach's alphas proved a good consistency (negative urgency alpha = 0.78, positive urgency alpha = 0.70, lack of premeditation alpha = 0.79, lack of perseverance alpha = 0.84, sensation‐seeking alpha = 0.83).

### 2.8 Questionnaire

A questionnaire for demographic data collection regarding age, gender, socio‐demographic category, and other elements was built. For other dimensions, the online form contained several questions with a Likert scale option for responses going from 1 to 7, (negative = 1, positive = 7). Participants were asked about their sense of loneliness during quarantine and their perceived impact on relational and emotional lives. They were also asked about the number of people they lived with during the quarantine (number of cohabitants) and the number of people they were in contact with every week (number of contacts), from colleagues to friends and family, regarding real‐life connections or virtual conversation. Precise questions were stated as listed here: (a) Have you ever been treated for psychological problems (with a psychologist or psychiatrist)? (yes/no), (b) Are you currently accompanied by a psychology professional (psychiatrist, psychologist, psychological care structure such as daycare/hospital/help association)? (yes/no), (c) Do you feel more isolated/lonelier at the moment (since the confinement)? (no, not at all = 1, yes, very often = 5), (d) Since the confinement, with how many people did you have regular contact (at least twice a week, either face to face or via telephone or video‐conference)? (e) How many people do you live with during confinement? (f) What is the overall impact of the confinement on your relationship life? (negative = 1, positive = 5), (g) What is the overall impact of the lockdown on your emotional life? (negative = 1, positive = 5).

### 2.9 Statistics

All measures used in our sample are normally distributed. Parametric tests computed with IBM SPSS Statistics (version 28.0.1.0 (142)). Pearson correlations analysis with 0.95% confidence intervals, for independent samples t‐test comparison (two‐sided), and linear regression. We used JAMOVI (2.3.28.0) for mediation analysis (Medmod module). The level of significance is set a priori to *p* < 0.05 (*), *p* < 0.001 (**), and *p* < 0.001 (***) were performed. Our analysis was post‐hoc hypothesis driven ones that gender will influence the aggression and impulsivity levels of the participants. Our subgroup analysis of men and women was a prespecified.

## 3 Results

### 3.1 Descriptive Analysis

The mean age for men was 34.97 years (13.850) and 32.90 years (12.91) for women. Economic status was divided into craftsman professions (0.02% of men's total sample size; 0.02% women's total sample size), executive jobs (0.22% men's total sample size; 0.125% women's total sample size), employees (0.31% men's total sample size; 0.25% women's total sample size), students (0.22% men's total sample size; 0.31% women's total sample size), low‐income class (0.08% men's total sample size; 0.12% women's total sample size), unemployed or retired (0.14% men's total sample size; 0.14% women's total sample size) and the rest being in “Other” category (dedicated to respondents that did not found themselves in any another category or did not wish to disclose). Looking at subgroup experiences with psychological care, 60 male participants (63.1% of men's total sample size) had no experience with psychological care, while 147 females (51.4% of women's total sample size) were in the same situation. At the time of the study, 85.5% of the male sample and 81.25% of the female one still not having psychological care.

T‐test comparison across male and female samples are recalled in the Table 1. Men (*n* = 95) and women (*n* = 286) mean were different for Isolation (*p* = 0.05, Cohen's *d* = 0.247), number of persons confined with (*p* = 0.007, Cohen's *d* = 0.345), relational life (*p* = 0.05, Cohen's *d* = 0.246), HADS‐A (*p* < 0.001, Cohen's *d* = 0.516), Negative Urgency (*p* < 0.001, Cohen's *d* = 0.464), Positive Urgency (*p* < 0.001, Cohen's *d* = 0.437), Lack of Premeditation (*p* = 0.003, Cohen's *d* = 0.356).

**Table 1:** Independent samples *t*‐test.

	*t*	df	Two‐sided *p*	Cohen's *d*	95% Confidence interval for Cohen's *d*	
					Lower	Upper
Age	−1.447	383	0.149	−0.171	−0.403	0.061
Isolation	1.937	330	0.054	0.247	−0.004	0.498
Number of contacts	−0.078	332	0.937	−0.010	−0.260	0.240
Number per. confined with	2.703	332	0.007	0.345	0.093	0.596
Relation. life	1.924	330	0.055	0.246	−0.006	0.497
Emotion. life	−0.423	309	0.673^a^	−0.057	−0.324	0.209
Sbqr	0.864	383	0.388	0.102	−0.130	0.334
HADS‐A	4.362	383	< 0.001	0.516	0.281	0.750
HADS‐D	0.485	383	0.628	0.057	−0.174	0.289
Negative urgency	3.924	383	< 0.001	0.464	0.230	0.698
Positive urgency	3.697	383	< 0.001	0.437	0.203	0.671
Lack of premeditation	3.014	383	0.003	0.356	0.123	0.589
LackPers	−1.822	383	0.069	−0.215	−0.447	0.017
Sensation seeking	−0.147	383	0.883	−0.017	−0.249	0.214
BHS	−1.169	383	0.243	−0.138	−0.370	0.094
AQ12	0.996	383	0.320	0.118	−0.114	0.349

*Note:* Student's *t*‐test.

Abbreviations: Anx, anxiety; AQ12, aggression score; Dep, depression; Emotion. Life, impact on emotional; H, hopelessness; Isolation, perceived sense of isolation, of being lonely during the quarantine; LackPers, lack of perseverance; LackPrem, lack of premeditation; Number contact, number of persons being in contact with during quarantine period; Number per. Confined with, number of persons being confined with during quarantine; Relation. Life, impact on relational life; Sbqr, score of Sbqr Scale.

^a^ Levene's test is significant (*p* < 0.05), suggesting a violation of the equal variance assumption.

### 3.2 Correlation Analysis

The statistical analysis revealed the correlation of all measured dimensions of psychopathology with HADS‐A and HADS‐D for each gender (See Table 2) and their correlation to suicidal risk through the SBQ‐R correlations array (see Table 2).

**Table 2:** Pearson's correlation table recalling correlation with HADS‐A and HADS‐D.

			Isolation	Relational life	Numb. isolated with	Emotional life	SBQ‐*r*	Negative urgency	Positive urgency	Lack of premeditation	Lack of perseverance	Sensation seeking	BHS	AQ12
Men	SBQ‐r	Pearson's *r*	0.151	−0.107	−0.053	−0.080	—	0.137	0.054	0.277	0.196	−0.005	0.602	0.195
		*p* value	0.172	0.336	0.632	0.503	—	0.180	0.598	**0.006**	**0.054**	0.958	**< 0.001**	**0.056**
		95% CI Upper	0.355	0.111	−0.241	0.154	—	0.328	0.251	0.451	0.381	0.194	0.716	0.379
		95% CI Lower	−0.067	−0.315	0.161	−0.306	—	−0.064	−0.147	0.082	−0.003	−0.205	0.458	−0.005
	HADS‐A	Pearson's *r*	0.275	−0.175	0.079	−0.274	0.362	0.161	0.158	0.305	0.106	−0.236	0.504	0.374
		*p* value	**0.012**	0.113	0.476	**0.020**	**< 0.001**	0.115	0.121	**0.002**	0.300	**0.020**	**< 0.001**	**< 0.001**
		95% CI Upper	0.463	0.042	−0.100	−0.045	0.524	0.349	0.347	0.475	0.299	−0.038	0.639	0.534
		95% CI lower	0.063	−0.377	0.252	−0.476	0.176	−0.039	−0.042	0.112	−0.095	−0.416	0.339	0.188
	HADS‐D	Pearson's *r*	0.223	−0.147	0.004	−0.285	0.347	0.368	0.040	0.298	0.328	−0.064	0.562	0.343
		*p* value	**0.043**	0.185	0.973	**0.015**	**< 0.001**	**< 0.001**	0.697	**0.003**	**0.001**	0.534	**< 0.001**	**< 0.001**
		95% CI upper	0.419	0.071	−0.213	−0.057	0.511	0.529	0.238	0.470	0.495	0.137	0.684	0.507
		95% CI lower	0.008	−0.351	0.211	−0.485	0.159	0.182	−0.161	0.105	0.137	−0.260	0.408	0.154
Women	SBQ‐*r*	Pearson's *r*	0.262	−0.078	−0.124	−0.219	—	−0.019	0.046	0.046	0.173	0.086	0.472	0.394
		*p* value	**< .001**	0.221	**0.049**	**< .001**	—	0.747	0.436	0.436	**0.003**	0.147	**< 0.001**	**< 0.001**
		95% CI upper	0.374	0.047	−0.235	−0.094	—	0.097	0.161	0.161	0.283	0.199	0.557	0.487
		95% CI lower	0.142	−0.200	−0.003	−0.336	—	−0.134	−0.070	−0.070	0.059	−0.030	0.377	0.292
	HADS‐A	Pearson's *r*	0.299	−0.278	0.085	−0.351	0.348	0.144	0.204	0.162	0.155	−0.010	0.429	0.507
		*p* value	**< 0.001**	**< 0.001**	0.182	**< 0.001**	**< 0.001**	**0.015**	**< 0.001**	**0.006**	**0.008**	**0.868**	**< 0.001**	**< 0.001**
		95% CI upper	0.408	−0.159	−0.037	−0.234	0.446	0.255	0.312	0.273	0.266	0.106	0.519	0.588
		95% CI lower	0.181	−0.389	0.212	−0.457	0.242	0.029	0.091	0.047	0.040	−0.125	0.330	0.416
	HADS‐D	Pearson's r	0.359	−0.354	0.000	−0.381	0.401	0.026	0.130	0.127	0.264	−0.051	0.547	0.386
		*p* value	**< 0.001**	**< 0.001**	0.998	**< 0.001**	**< 0.001**	**0.660**	**0.028**	**0.031**	**< 0.001**	**0.392**	**< 0.001**	**< 0.001**
		95% CI upper	0.463	−0.241	−0.119	−0.267	0.494	0.141	0.242	0.239	0.369	0.065	0.623	0.480
		95% CI lower	0.246	−0.458	0.114	−0.485	0.300	−0.090	0.014	0.012	0.153	−0.165	0.460	0.283

*Note:* Student's *t*‐test.

Abbreviations: Anx, anxiety; AQ12, aggression score; CI, confidence interval; Dep, depression; H, hopelessness; Isolation, perceived sense of isolation, of being lonely during the quarantine; LackPers, lack of perseverance; LackPrem, lack of premeditation; Numb. isolated with, number of persons isolated with; Number contact, number of persons being in contact with during quarantine period; Number per. confined with, number of persons being confined with during quarantine; Relation. life, impact on relational life; emotion. life, impact on emotional; Sbqr, score of Sbqr Scale.

### 3.3 Regression Analysis

Linear regressions (single step) were performed using SPSS for HADS‐A with all correlated elements as predictive factors for men and women revealing BHS and AQ12 as predictors. More precisely, for men, BHS was a predictor (beta = 0.308*) and AQ12 (beta = 0.267*) with *R*² = 0.420 and *F* = 6.619. For women, BHS (beta = 0.216***) and AQ12 (beta = 0.344) were the predicting factors with *R*² = 0.402 and *F* = 15.675. For prediction of HADS‐D predictions based on correlated factors (reported in Table 2), the following results were obtained: for men, BHS was a predictor (beta = 0.402**) and negative urgency too (beta = 313**) with *F* = 6.911 *R*² = 0.467, but for women, relational life (beta = −0.158**); BHS (beta = 0.313***) and AQ12 (beta = 0.122*) with *R*² = 0.410 and *F* = 17.525.

Regarding the prediction of SBQ‐R scores, in the men's sample, from all the correlated factors (reported in Table 2), only BHS was a relevant factor (beta = 0.556***) with *F* = 13.420 and *R*² = 0.368 whereas in women's sample, sense of loneliness (beta = 0.174*), BHS (beta = 0.276***) and AQ12 (beta = 0.244***) where predictors with *F* = 13.632 and *R*² = 0.322.

### 3.4 Mediation Effect Exploration

**Figure 1:**
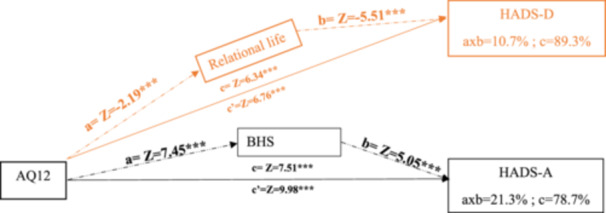
Mediations analysis. *p* < 0.001 = ***.

We tested mediation relations to highlight the effect of AQ12 on for HADS‐D, and HADS‐A (see Figure 1) with a mediating effect of respectively relational life, and BHS.

Reported results are significant (*p* < 0.05) based on the Sobel test.

## 4 Discussion

Our results revealed the comparable level of aggression for women and men, and its actual impact on anxiety and depression levels for women, and as a predictive factor for suicidal risk in this population. For women, the correlation array was much more complex for HADS anxiety and depression and SBQ‐R. Emotional and relational disturbances more influenced women compared to men.

These results look aligning with the recent literature where gender did not appeared to play a role in patient's aggression [42] even if presented in 22 articles hypothetizing the dominance of male aggression. For instance, our t‐test results on intra‐group comparison regarding previous experiences with psychological care showed that women did have differences in clinical dimensions (SBQR, HADS‐A, HADS‐D, and Lack of perseverance) and age and number of persons being isolated with. Moreover, males did have differences in suicidal risk, anxiety, hopelessness, and aggression. It appears that aggression is indeed a differentiating factor for psychological issues for men but not for women, even if aggression stands as a predictive factor. One needs to consider the impact of aggression on women's mental health issues, both in clinical and nonclinical samples.

However, some results regarding impulsivity disorders like attention deficit hyperactivity disorder (ADHD) are known to suffer from gender bias and referral bias, so studies on “male” dimensions like impulsivity and aggression could be impaired. For example, Fraticelli et al., 2022 [43] reported the underestimation of ADHD diagnosis for women pointing at the consequence for equity as they will be less likely to be referred for treatment than males with ADHD, and they encourage equal representation of both sexes in further studies.

To some extent, the correlation and causality relation between aggression and anxiety are important aspects of mental health in specific populations like adolescents. Chung et al. in 2019 [44] demonstrated that anxiety was associated with aggression scores. More recently, in 2021, Klimovich‐Mickael et al. [45] showed a relation between anxiety, anger, and physical aggression, with hostility and verbal aggression being more prevalent than other emotions.

Our results are congruent with the hypothesis of a need for a stronger multi‐agency or multi‐sectoral approach to suicide prevention, intervention, and postvention during COVID‐19‐related suicide cases [46, 47]. For example, in 2020, Wathelet et al. [48], risk factors encouraging mental health issues due to quarantine in the student sample were a variety of sociodemographic factors and gender: female gender or nonbinary, precariousness, history of psychiatric follow‐up, symptoms compatible with COVID‐19, social isolation, and low quality of the information received.

The results of the mediation analysis also question the need for the modelization of influencing factors of HAD‐D and HADS‐A as they suggest that AQ12 even if standing as a strong predictor, needs to be considered together with other clinical aspects (BHS) but also socio‐demographic aspects (relational life). These results are coherent with the literature pointing out the impact of social or environmental modifiers. For example, the general aggression model (2018) differentiates distal causes and processes (the biological, psychological, and social elements) and proximate causes (internal pathways of cognitive processes and decision‐making) that interact together [49]. It is recognized that anxiety can trigger and support the development of aggression [50], and be a mediator for aggression prediction [51] but based on our preliminary results, further research could look at the influence of aggression on anxiety. The same applies to depression where aggression is often consequential [50]. Moreover, these results being specific to the female gender, they encourage more extensive research in our consideration around “male.” For example, recent research showed that sex differences in adolescents for physical aggression were varying a lot across cultural contexts; contrary to expectations derived from social role theory, sex differences in physical aggression decrease as societal gender inequality increases, some countries having smaller sex differences in frequent fighting [52].

Our research contains several limitations. The first limitation is that our sample comprises more women than men; however, both samples were considered comparable [53]. Another limitation of our study may be that women are more prone to emotional and psychological identification and give more refined scores when answering the same questionnaires as men are supposed to be “brave” and women “emotional” [54]. Another limitation the author oversees is the sample selection bias as the internet recruitment procedure may not be representative of the overall French population. For example, the vast majority of the respondents did not precisely answer income‐related information in the socio‐demographic questions, making it difficult for us to see the potential for generalization of the results. The lack of generalization of our results can also be since impulsivity and aggression are highly culturally dependent so our results may only be representing the French context [55, 56]. However, social differences may be minimal across western European countries at the time as most of them were applying look‐alike quarantine [56, 57]. Another bias may arise from the social status of participants as some of them may have been working as care or social workers which were highly subjected to stress during COVID 19 and it could have played a role in gender differences as most of the care workers are demographically female [58, 59]. Additionally, our research focused on clinically measured based outcomes but should have been enhanced using stress level scores to refine the level of distress from “non to normal” to “severe” and then “pathological impactful.” However, the HADS scale being a first‐line used scale (endocrinology, psychiatry, somatic care), its use reflected the overall psychological distress of participants. Finally, even if controlling for the former or present use of professional psychological care, the present research didn't assess the extent of coping strategies that the participant was using at the time of the study. For example, one may have been using coping strategies, community support, self‐help, or other resources that may not be encompassed in the formulated demographic questions. Further research may include a coping strategy scale and specify the access to mental health resources (availability, format, duration, frequency, techniques, etc.). On the methodological level, the use of comparative analysis with sample sizes differing from each other can have advantages and disadvantages (small sample size that can create biases in certain analyses but still be valuable in clinical data analysis see [60, 61]. Our analysis did fulfill the requirements for properly comparing independent samples using a t‐test [62, 63] and is coherent with the applied psychology research sample [64]. Finally, as the procedure was based on declaration of fit for exclusion and inclusion criteria, the results are conditional to stated fit and not external clinical validation. Indeed, any further research would need to include more strict recruitment procedure.

## 5 Conclusion

In conclusion, further research is imperative to deepen the understanding of aggression and impulsivity dynamics in women, addressing a critical gap in recognizing gender‐based vulnerabilities. There is a clear need to adapt and expand aggression and impulsivity regulation programs beyond forensic settings [65], ensuring their applicability to both clinical and nonclinical populations. Recognizing the nuanced impact of aggression on women's mental health is vital, given its significant influence on anxiety, depression, and suicidal risk. Tailored interventions should prioritize relational and emotional disturbances that disproportionately affect women, while clinicians must be equipped to address gender biases in symptom reporting, ensuring equitable care for all patients. Comprehensive assessment models should integrate tools that evaluate aggression, impulsivity, and anxiety as interconnected factors, incorporating clinical and sociodemographic contexts. Frameworks like the General Aggression Model (2018) [66] offer valuable insights into the pathways linking these elements. Future studies must also explore gender dynamics in aggression and impulsivity across diverse cultural settings, paving the way for innovative, inclusive, and effective aggression‐regulation programs that extend to broader clinical applications.

## Author Contributions

**Sylvia Martin:** conceptualization, investigation, funding acquisition, writing–original draft, methodology, validation, visualization, writing–review and editing, software, formal analysis, project administration, data curation, supervision, resources. All authors have read and approved the final version of the manuscript Sylvia Martin had full access to all of the data in this study and takes complete responsibility for the integrity of the data and the accuracy of the data analysis.

## Consent

The author have nothing to report.

## Ethics Statement

The ethical approval is deemed unnecessary in French context as the study falls into the “Jardé Law” RIPH.^1^ Three for noninterventional research (*Code of Public Health, Articles L1121‐1 à L1128‐12*). All participants gave online informed consent to participate in this experiment for experiments involving human subjects. All the experiments in this study were conducted according to ethical provisions from the Psychologists and World Medical Association Code of Ethics (Helsinki Declaration).

## Conflicts of Interest

The author declares no conflicts of interest.

## Transparency Statement

The lead author Sylvia Martin affirms that this manuscript is an honest, accurate, and transparent account of the study being reported; that no important aspects of the study have been omitted; and that any discrepancies from the study as planned (and, if relevant, registered) have been explained.

## Data Availability

Data will be available upon reasonable request from the corresponding author (S.M.).

## Endnotes

1 Stands for “Research organized and conducted on healthy or ill volunteers with the aim of advancing biological or medical knowledge.” For the French “Recherche Impliquant la Personne Humains” directly translating as Research Involving Human Persons.
